# The Difficulty of Diagnosing Kikuchi-Fujimoto Disease in Infants and Children Under Six Years Old: Case Report and Literature Review

**DOI:** 10.7759/cureus.7383

**Published:** 2020-03-24

**Authors:** Yasuji Inamo

**Affiliations:** 1 Pediatrics, Nihon University School of Medicine, Tokyo, JPN

**Keywords:** kikuchi-fujimoto disease, hemophagocytic lymphohistiocytosis, young child, serum alkaline phosphatase, interstitial pneumonia, mediastinal lymph nodes

## Abstract

We came across a 20-month-old boy with Kikuchi-Fujimoto disease (KFD) who showed atypical symptoms that were difficult to diagnose. His symptoms were different from those experienced in common clinical KFD cases. Hence, we report his case presentation and review the literature on the difference in KFD symptoms between infants and young children (under six years of age), and school-age children (6 to 20 years).

A 20-month-old boy was admitted to our hospital because of prolonged fever and an enlarged right axillary lymph node with pain. He developed erythema, which was like rubella, on the face, trunk, and upper and lower extremities. The rash soon disappeared after two days. The cervical lymph nodes were not palpable. Hepatomegaly and splenomegaly were palpable. Leukopenia and a decrease in platelets were seen in the blood count. Curiously, leukocytosis developed after leukopenia was first observed. Serology titers for several pathogens were negative. A CT scan of the lungs showed swelling of the mediastinal lymph nodes and interstitial pneumonia. The examination of a specimen of the axillary lymph node obtained by biopsy was compatible with KFD.

English language reports on KFD were reviewed. Fourteen (14/335: 4.2%) cases in children under six years in addition to the current case and 321 (321/335: 95.8%) cases of school-age children (6-20 years) were found in the literature. Children of school age tend to more commonly be afflicted with KFD, which is characterized by pyrexia, leukopenia, and cervical lymphadenopathy with tenderness. Inversely, major symptoms of KFD patients under six years old were lesions of the lungs in three cases, leukocytosis in six cases, and generalized lymphadenopathy in eight cases, in contrast with symptoms of school-age children.

It is concluded that leukocytosis, generalized lymphadenopathy in sites other than the cervical lymph nodes, and lesions of the lungs are characteristic symptoms of severe KFD in patients under six years old, for whom the occurrence is very rare.

## Introduction

Kikuchi-Fujimoto disease (KFD) was first reported in Japan [1]. It is a self-limiting and benign disease most often seen in young women, and it is characterized by pyrexia, leukocytopenia, and cervical lymphadenopathy with tenderness. KFD can also be seen in children, especially boys, over 10 years old, and most cases have been reported in Asian countries [2]. KFD is very rare in infants and young children (under six years of age). Furthermore, it is difficult to diagnose in this age group because very young patients do not show the classical signs and symptoms of KFD.

Our experience with a 20-month-old boy with KFD led us to review the differences in clinical features between KFD patients under six years of age and patients of school-age (6 to 20 years).

As there is no specific and diagnostic examination for KFD, physicians must first remind themselves of KFD from clinical features. The final diagnosis is only made by incisional lymph node biopsy. Furthermore, it is very difficult to diagnose because the onset of KFD rarely occurs in children under six years old.

Although fatal and severe cases have been reported in infants and young children with KFD, there has been no report about the difference from symptoms of KFD after school age.

Our case report aims to present clinical features of a rare case of a 20-month-old boy with KFD and to report symptoms of KFD in infants and young children in the literature.

## Case presentation

A 20-month-old boy was admitted to another hospital because of fever (body temperature up to 39.1°C) continuing for seven days. Eight days before admission, the boy had an enlarged right axillary lymph node spreading to the chest wall with pain. Treatment with ampicillin and clindamycin was ineffective for the fever and enlarged axillary lymph node. Two days later, he developed erythema, which was like rubella, on the face, the trunk, and the upper and lower extremities. The rash soon disappeared after two days. The patient had no history of allergy, travel, or exposure to sick persons. His family did not breed any animals. Because of his persistent fever, the boy was referred to our hospital, and laboratory studies were performed.

On examination at admission, a firm, tender right anterior axillary lymph node, 3.5 cm in diameter, was palpable. The cervical lymph nodes were not palpable, and there was no overlying erythema or warmth. Hepatomegaly and splenomegaly were palpable. The blood counts were as follows: hemoglobin 10.2 g/dL, leukocytes 2,400/µL (neutrophils 40.2%, lymphocytes 50.8%, and monocytes 2.0%), and platelets 13.0×10^4^/µL. However, leukocytes had increased to 13,510/µL in the previous 14 days. The erythrocyte sedimentation rate was 32 mm/hour. The level of aspartate aminotransferase was 87 IU/L (normal range: 11-33 IU/L), alanine aminotransferase was 55 IU/L (normal range: 6-43 IU/L), lactate dehydrogenase was 571 IU/L (normal range: 120-245 IU/L), alkaline phosphatase was 843 IU/L (normal range: 430-1,200 IU/L, see the details below), and C-reactive protein was 0.11 mg/dL (normal range: less than 0.15 mg/dL). Serology titers for Epstein-Barr virus, cytomegalovirus, Bartonella henselae, herpes simplex virus, Toxoplasma, adenovirus, and HIV were negative. A Mantoux test was negative after 48 hours. Anti-nuclear antibody was negative. Bone marrow examination revealed normocellular marrow.

**Figure 1:**
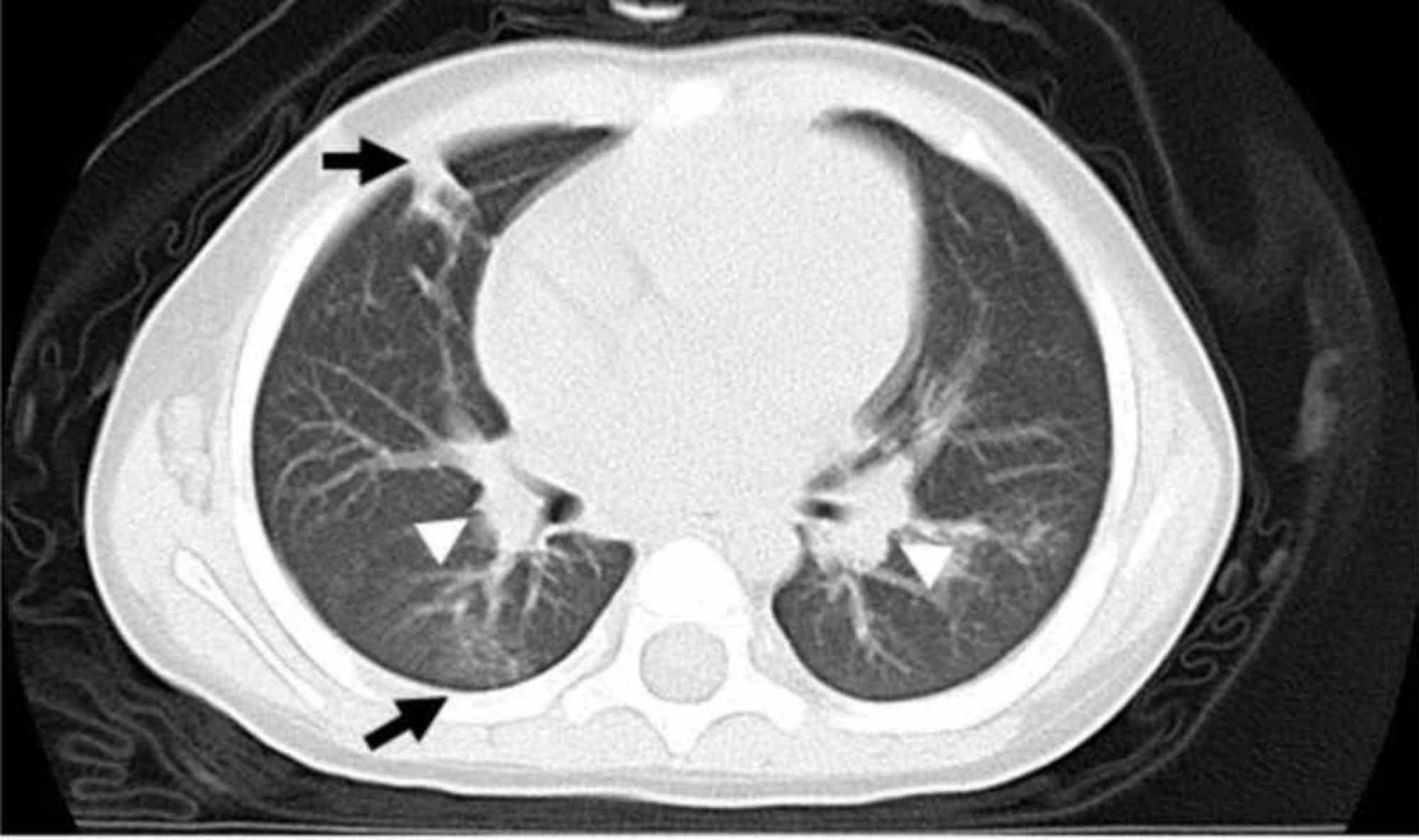
CT Scan of the Lungs Axial image from a scan with lung windows shows faint ground-glass and micronodular opacities in the lower lungs and swelling of both mediastinal lymph nodes (white head arrow). The ground-glass opacities have a marked peribronchovascular distribution in the lower right lung (arrow).

A computed tomography (CT) scan of the lungs showed swelling of the mediastinal lymph nodes and interstitial pneumonia, accompanied by scattered smaller lymph nodes in both lung fields (Figure 1).

**Figure 2:**
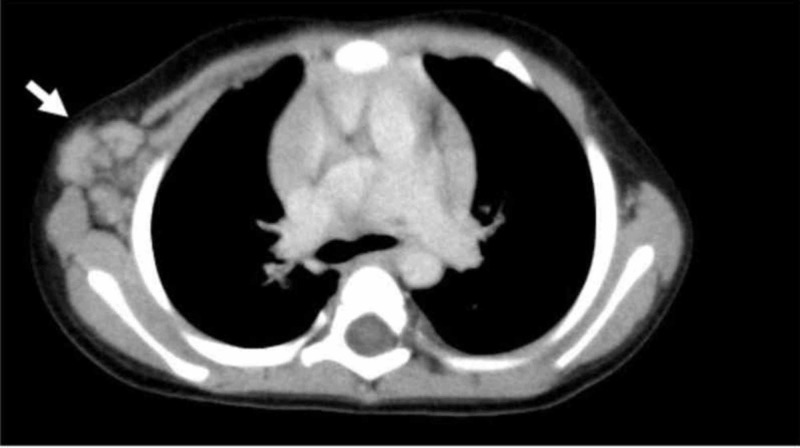
CT Scan of the Chest Wall Axial image from a scan with mediastinal windows discloses swelling of the right lymph nodes and their spreading along the chest wall (arrow).

A CT scan of the thorax disclosed right axillary lymph nodes and swelling of the many thoracic wall lymph nodes (Figure 2). A CT scan of the abdomen and pelvis showed swelling of the mesenteric lymph nodes and inguinal lymph nodes. The results of cardiac ultrasonography were normal, with no aneurysms.

**Figure 3:**
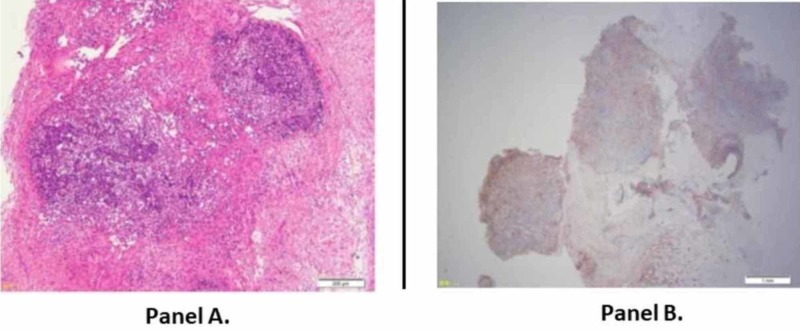
Biopsy Specimen of the Axillary Lymph Node Panel A: Histopathology shows lymphoid follicular areas containing lymphoid cells and histiocytes, and paracortex areas with coagulative necrosis. There is no neutrophilic or eosinophilic infiltration. Axillary lymph node specimen contains lymphoid cells and debris. A structure of lymphoid follicles is moderately destroyed (hematoxylin and eosin staining; magnification, ×40). Panel B: Immunohistochemistry shows a predominance of positive cells for the histiocytic marker CD68 in the lymphoid follicle (original magnification, ×90).

Blood, sputum, throat swab, and stool cultures were negative. The examination of a section of the axillary lymph node obtained by biopsy was compatible with KFD (Figure 3). His parents gave written informed consent for the biopsy. Recovery was observed after the excision of the lymph node, and no further specific treatment was required. The fever continued for 30 days in his clinical course overall.

## Discussion

Our 20-month-old patient had prolonged fever and a markedly enlarged lymph node, which was like a bacterial abscess along the chest wall because of accompanied leukocytosis. It was unanticipated that we would find interstitial pneumonia and swelling of bilateral hilar lymph nodes and mesenteric lymph nodes despite no cervical lymphadenopathy being revealed by CT of the lungs and abdomen. His manifestations were much more atypical than those of typical KFD and difficult to diagnose. It was impossible to make a diagnosis until we performed an incisional biopsy of the axillary lymph node. Because this biopsy provided the only diagnosis, it is necessary to develop a supplemented diagnostic test of KFD in the future.

As the number of patients under six years old with KFD is very small, it is a diagnostic challenge to discriminate KFD from many febrile illnesses. We, therefore, reviewed the literature to identify and describe such cases. We defined the specific cut-off of under six years of age as the criterion for “infants and young children” for the KFD literature review.

Literature review

We searched the PubMed database for English articles related to KFD in patients under six years of age. A date range was set from 1982 to 2016. The last search update was performed on February 9, 2016. The terms “Kikuchi Fujimoto disease” and “child or infant or review or histiocytic necrotizing lymphadenitis or hemophagocytic lymphohistiocytosis” were used as the keywords. Screening references of retrieved articles identified some publications. Seventeen publications were retrieved [2-19]. Fourteen cases were identified [3, 8, 10-16, 18]. Patients with KFD under six years old are very rare (n=14, age range: 9 months to 5 years). The number of patients with KFD approximately increases after school age.

Result of the literature review

A total of 355 children (age range: 9 months to 20 years) were identified as having KFD in the literature. Of these, 14 patients (14/335: 4.2%) were under six years old (age range of 9 months to 5 years). Patients aged six and over (age range: 6-20 years) numbered 321 (321/335: 95.8%), of whom 40 (40/145: 27.1%) had leukocytopenia, two (2/68: 2.8%) had leukocytosis, one had chest deformity, 10 had generalized lymphadenopathy (including superficial, axillary, mesenteric, and mediastinal lymph node swelling), 25 had hepatosplenomegaly and hepatomegaly, 32 had a skin rash, four (4/38: 10.5%) had hemophagocytic lymphohistiocytosis (HLH) with KFD, and none were terminal.

The clinical manifestations were obtained from 10 of the 14 patients identified as under six years old (age range: 9 months to 5 years). The 10 cases with detailed descriptions of clinical features are shown in Table 1. Of these patients, seven were male; two had leukocytopenia; six had leukocytosis; three had bronchial, lung, and pleural lesions; two had generalized lymphadenopathy (including superficial, axillary, mesenteric, and mediastinal lymph node swelling); one had hepatosplenomegaly and hepatomegaly; two had a skin rash; one (1/14: 7.1%) had HLH with KFD; and one died (Table 1).

**Table 1:** Demographic, Clinical, and Laboratory Data of Ten Patients Under Six Years Old with Kikuchi-Fujimoto Disease ND: not described

Parameter	Lee BC, USA [10] (n=10)	Kim TY, Korea [11] (n=40)	Chuang CH,, Taiwan [12] (n=64)	Zou CC , China [13] (n=36)	O’Neill D, UK [18] (n=1)	Han HJ, Korea [3] (n=15)		Yoo LH, Korea [4] (n=33)	Kim HS, Korea [16[ (n=1)	Our case
Age, months (sex)	22mo.(male)	24 mo. (male)	24mo. (male)	17 mo.(gender ?)	19 mo. (Male)	2yr (female)	5yr (female)	9 mo. (male)	9 mo. (male)	20 mo. male
Leukocytosis(>10,000/) /leukocytopenia	leukocytosis	leukocytosis	ND	leukopenia or WNL? but leukocytosis(-)	ND	leukocytosis	leukopenia	ND	leukocytosis	leukopenia and leukocytosis
Main lesion of lymphadenopathy	Cervical/supraclavicular lymphadenopathy	cervical lymphadenopathy	ND	ND	cervical lymphadenopathy	cervical lymphadenopathy	cervical lymphadenopathy	ND	cervical lymphadenopathy	right axillary lymphadenopathy
Prolonged fever	ND	ND	a prolonged fever for 42 days	ND	a prolonged fever for 12 days	a prolonged fever for 10 days	a prolonged fever for 26 days	ND	a prolonged fever for 10 days	a prolonged fever for 30 days
Hepatomegaly /splenomegaly /HLH	ND	ND	ND	ND	ND	HLH	ND	Hepatomegaly (+)	ND/ND/HLH before 5 months the onset of KFD	Hepatomegaly (+) Splenomegaly (+)
Generalized lymphadenopathy	ND	(-)	ND	ND	ND	ND	ND	ND	the supraclavicular region, mediastinum, retroperitoneum, pelvis and inguinal sites	(+) the mediastinum, mesentery, groin
Lungs /Heart involvement	bronchial wall thickening	ND	ND	ND	pleural effusion (+) pericardial effusion (+)	ND	ND	ND	ND	interstitial pneumonia
Erythema	(+)	ND	ND	ND	ND	ND	ND	ND	ND	(+)
Outcome	recovery	recovery	recovery	recovery	Died	recurrence	recovery	recurrence Perinatal CMV infection	recovery	recovery

Note: Percentages are given where the total number (as a denominator) was confirmed in the literature. Only the number of cases is given when the total number was unclear from the references.

Limitations

The major limitation of the present study is the possibility of incomplete extraction of clinical features from the retrieved literature. It should be noted that such data might underestimate the true prevalence of clinical features in patients with KFD because of a lack of descriptive information. All potential sources of bias and confounders cannot be excluded from any study.

Although the small sample size prevents a statistical analysis of the leukocyte count, it tended to leukocytosis under six years, but leukopenia occurred in only a few cases. Curiously, we experienced in our case that leukopenia inversely changed to leukocytosis during the clinical course. Leukopenia is common in KFD. However, it is difficult to diagnose KFD by leukocytosis because it mimics infectious diseases. The patients aged six or over (age range: 6-20 years) tend to have leukopenia rather than leukocytosis. Inversely, five (5/6: 83.3%) of the patients under six years old (age range: 9 months to 5 years) had leukocytosis, and only two (2/6: 25%) of the patients under six years old had leukopenia.

Although the pathogenesis of interstitial pneumonia in KFD is unclear, we found reports of four adult patients: a 32-year-old man with interstitial pneumonia and pleurisy; a 33-year-old man with cryptogenic organizing pneumonia; a 33-old-year woman with interstitial pneumonia; and a 21-year-old woman with deadly interstitial pneumonia, pleurisy, and pericardial effusion.

Three cases of children under six years old presented bronchial wall thickness, pleurisy, and interstitial pneumonia (our case). Conversely, one (1/335: 0.3%) of the patients aged six years or over (age range: 6-20 years) had a chest deformity (not described in detail). Although only a few cases of lung disease accompanied KFD, it is better to carry out CT of the lungs in children under six years old.

It is possible that KFD without painful cervical lymphadenopathy greatly delays diagnosis, as in our case. However, KFD without painful cervical lymphadenopathy is not always a specific symptom of KFD in children under six years old. Three (3/218: 1.4%）of the patients aged six years and over (age range: 6-20 years) and one (1/8: 0.13%) of the patients under six years old (age range: 9 months to 5 years) had no painful cervical lymphadenopathy.

KFD tends to be accompanied by HLH, which is an important disease to discriminate it from febrile illnesses. KFD occurring with HLH is severe, but it is not entirely specific under six years of age: four (4/38: 10.5%) of the patients aged six years and over (age range of 6-20 years) and one (1/14: 7.1%) of the patients under six years old (age range: 9 months to 5 years) had complications with HLH. Furthermore, it is also necessary to note that KFD accompanied by HLH can be caused by other disorders, such as juvenile myelomonocytic leukemia or juvenile idiopathic arthritis [19].

**Figure 4:**
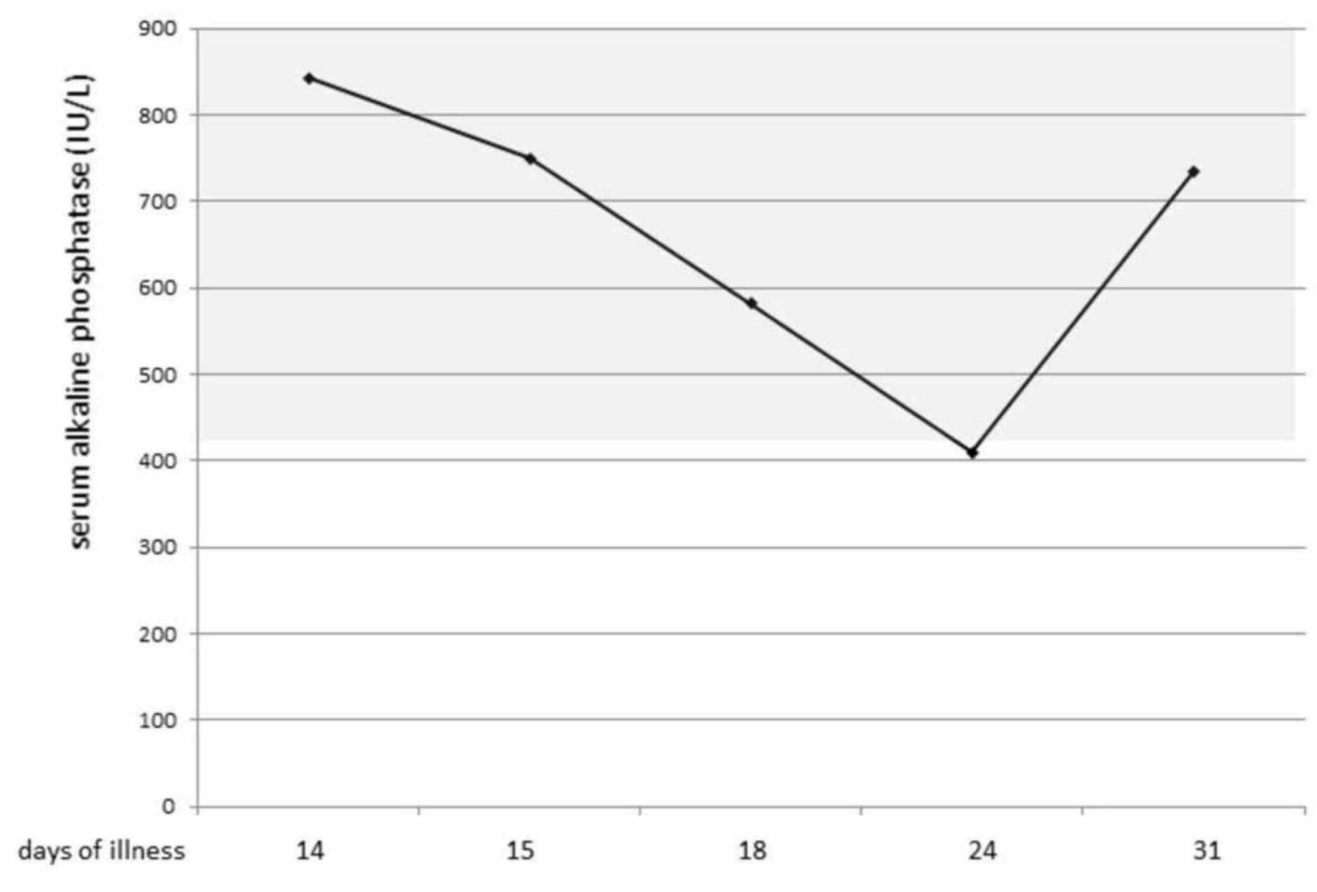
Changes in the Levels of Serum Alkaline Phosphatase in Our KFD Patient The patient's levels of serum alkaline phosphatase continued to decrease during the acute phase of KFD and returned to normal levels toward convalescence.

It is important to develop a KFD-specific diagnostic examination, but currently, there is no supplemental diagnostic test for KFD. Particularly, we recommend the measurement of serum alkaline phosphatase levels to diagnose KFD because the levels of serum alkaline phosphatase continue to decrease during KFD. This phenomenon is a cue for suspecting KFD. Our patient also showed a decrease in serum alkaline phosphatase (Figure 4)[20].

As mentioned above, we should suspect KFD despite its rarity when we examine a young child with prolonged fever, leukocytosis, and painful superficial lymphadenopathy despite no painful cervical lymph node. In addition, it is necessary to further examine the whole body because of previously reported cases of KFD accompanied by two and more lesions on multiple organs.

## Conclusions

KFD is rare in children under six years old, and it presents various symptoms. In this age group, it tends to present leukocytosis rather than leukopenia. KFD in children under six years is usually complicated with lesions of multiple organs, such as interstitial pneumonia or hepatosplenomegaly or both. When KFD is suspected, testing for a decrease in levels of serum alkaline phosphatase is a useful clue.

## References

[1] Kikuchi M (1972). Lymphadenitis showing focal reticulum cell hyperplasia with nuclear debris and phagocytes: a clinicopathological study. Nippon Ketsueki Gakkai Zasshi.

[2] Lin HC, Su CY, Huang SC (2005). Kikuchi's disease in Asian children. Pediatrics.

[3] Han HJ, Lim GY, Yeo DM, Chung NG (2009). Kikuchi's disease in children: clinical manifestations and imaging features. J Korean Med Sci.

[4] Yoo IH, Na H, Bae EY, Han SB, Lee SY, Jeong DC, Kang JH (2014). Recurrent lymphadenopathy in children with Kikuchi-Fujimoto disease. Eur J Pediatr.

[5] Yu HL, Lee SS, Tsai HC (2005). Clinical manifestations of Kikuchi's disease in southern Taiwan. J Microbiol Immunol Infect.

[6] Wang TJ, Yang YH, Lin YT, Chiang BL (2004). Kikuchi-Fujimoto disease in children: clinical features and disease course. J Microbiol Immunol Infect.

[7] Park HS, Sung MJ, Park SE, Lim YT (2007). Kikuchi-Fujimoto disease of 16 children in a single center of Korea. Pediatr Allergy Immunol.

[8] Lee KY, Yeon YH, Lee BC (2004). Kikuchi-Fujimoto disease with prolonged fever in children. Pediatrics.

[9] Lin HC, Su CY, Huang CC, Hwang CF, Chien CY (2003). Kikuchi's disease: a review and analysis of 61 cases. Otolaryngol Head Neck Surg.

[10] Lee BC, Patel R (2013). Kikuchi-Fujimoto disease: a 15-year analysis at a children's hospital in the United States. Clin Pediatr (Phila).

[11] Kim TY, Ha KS, Kim Y, Lee J, Lee K, Lee J (2014). Characteristics of Kikuchi-Fujimoto disease in children compared with adults. Eur J Pediatr.

[12] Chuang CH, Yan DC, Chiu CH (2005). Clinical and laboratory manifestations of Kikuchi's disease in children and differences between patients with and without prolonged fever. Pediatr Infect Dis J.

[13] Zou CC, Zhao ZY, Liang L (2009). Childhood Kikuchi-Fujimoto disease. Indian J Pediatr.

[14] Supari D, Ananthamurthy A (2014). Kikuchi-fujimoto disease: a study of 24 cases. Indian J Otolaryngol Head Neck Surg.

[15] Kang HM, Kim JY, Choi EH, Lee HJ, Yun KW, Lee H (2016). Clinical characteristics of severe histiocytic necrotizing lymphadenitis (Kikuchi-Fujimoto disease) in children. J Pediatr.

[16] Kim HA, Im SA, Chung NG, Kang JH, Park GS (2011). Disseminated Kikuchi disease associated with hemophagocytic syndrome in an infant: whole-body MRI. Indian J Pediatr.

[17] Ramanan AV, Wynn RF, Kelsey A, Baildam EM (2003). Systemic juvenile idiopathic arthritis, Kikuchi's disease and haemophagocytic lymphohistiocytosis--is there a link? Case report and literature review. Rheumatology (Oxford).

[18] O'Neill D, O'Grady J, Variend S (1998). Child fatality associated with pathological features of histiocytic necrotizing lymphadenitis (Kikuchi-Fujimoto disease). Pediatr Pathol Lab Med.

[19] Gerritsen A, Lam K, Marion Schneider E, van den Heuvel-Eibrink MM (2006). An exclusive case of juvenile myelomonocytic leukemia in association with Kikuchi's disease and hemophagocytic lymphohistiocytosis and a review of the literature. Leuk Res.

[20] Inamo Y (2017). Low serum alkaline phosphatase activity in Kikuchi-Fujimoto disease. Medicine (Baltimore).

